# Adaptive noise depression for functional brain network estimation

**DOI:** 10.3389/fpsyt.2022.1100266

**Published:** 2023-01-10

**Authors:** Di Ma, Liling Peng, Xin Gao

**Affiliations:** ^1^College of Information Science and Technology, Nanjing Forestry University, Nanjing, China; ^2^Department of PET/MR, Shanghai Universal Medical Imaging Diagnostic Center, Shanghai, China

**Keywords:** Autism spectrum disorder, functional brain network, Pearson's correlation, adaptive noise depression, functional magnetic resonance imaging

## Abstract

Autism spectrum disorder (ASD) is one common psychiatric illness that manifests in neurological and developmental disorders, which can last throughout a person's life and cause challenges in social interaction, communication, and behavior. Since the standard ASD diagnosis is highly based on the symptoms of the disease, it is difficult to make an early diagnosis to take the best cure opportunity. Compared to the standard methods, functional brain network (FBN) could reveal the statistical dependence among neural architectures in brains and provide potential biomarkers for the early neuro-disease diagnosis and treatment of some neurological disorders. However, there are few FBN estimation methods that take into account the noise during the data acquiring process, resulting in poor quality of FBN and thus poor diagnosis results. To address such issues, we provide a brand-new approach for estimating FBNs under a noise modeling framework. In particular, we introduce a noise term to model the representation errors and impose a regularizer to incorporate noise prior into FBNs estimation. More importantly, the proposed method can be formulated as conducting traditional FBN estimation based on transformed fMRI data, which means the traditional methods can be elegantly modified to support noise modeling. That is, we provide a plug-and-play noise module capable of being embedded into different methods and adjusted according to different noise priors. In the end, we conduct abundant experiments to identify ASD from normal controls (NCs) based on the constructed FBNs to illustrate the effectiveness and flexibility of the proposed method. Consequently, we achieved up to 13.04% classification accuracy improvement compared with the baseline methods.

## 1. Introduction

Autism spectrum disorder (ASD) is one of the most common psychiatric illnesses characterized by repetitive behaviors and persistent impairments in communication and interaction (1, 2). Referring to a current report provided by the CDC of the USA (3), the overall ASD prevalence is rapidly increasing, rising from 6.7 per 1,000 children aged 8 years in surveillance years 2000 and 2002 to 23.0 in the surveillance year 2018. However, doctor's diagnosis of ASD highly depends on people's developmental history and behavior, which may result in delayed final diagnosis and thus missed early help (4). To get an early diagnosis, gene-level measurements can help, but their high cost and complexity impedes the spread (5, 6). Recently, researchers have shown that unusual brain activity and abnormal functional disruptions in brain regions highly correlate with ASD, making it capable to discover informative biomarkers and analyze brain activity to help diagnose ASD (7–9).

As a successful non-invasive technique for measuring brain activity, functional magnetic resonance imaging (fMRI) has been successfully used to aid in the early diagnosis of ASD (10–12). Functional brain network (FBN) is one of the most popular tools to help diagnosis based on the fMRI data (13–15). Generally, FBN is constructed by the brain regions of interest (ROIs) and their correlations, such as the statistical dependence between different ROIs (16, 17). Compared to the current study, which directly utilizes the fMRI data to identify the ASD from normal controls (NCs), the FBN-based methods can provide more stable measurements among neural time series of the brain that highly relates to some neurological diseases, including ASD, mild cognitive impairment (MCI) (18), Alzheimer's disease (AD) (19), and chronic tinnitus (CT) (20).

Second-order statistics are most commonly used to estimate FBN, and typical models include Pearson's correlation (PC) (21) and sparse representation (SR) (22). PC enjoys an efficient and robust FBN estimation that captures the full correlation among ROIs, but it has dense connections and is affected by confounding effects from other brain regions (16). Instead, SR can capture the partial correlation that eliminates the potential effects of other brain regions. However, the computation of the inverse covariance matrix is involved in partial correlation, which is ill-posed (23). Therefore, SR is equipped with an L1-norm regularizer to obtain a more stable partial correlation, leading to relatively lower computation efficiency than PC.

Despite their successful applications, the existing methods rarely take into account the data acquisition noise, which usually leads to poor FBN estimation and thus poor performance on disease identification. Before FBN estimation, the data preprocessing follows a standard pipeline to avoid influence caused by noisy signals (24), which is still not easy to filter out all the artifacts/noises from the data due to the weak fMRI signals. For example, some preprocessing steps may further introduce the notorious noisy time points into the data (e.g., spatial normalization) (25).

To address the earlier issues, in this study, we propose a novel FBN estimation strategy by embedding a noise modeling term to depress the effects of noise on FBN estimation. The noise term measures the noises and their correlation among time series to capture the noise pattern implied in time series. We show that such a term appears to be a modification of the data-fitting term, which has a great influence on FBN estimation. Then we introduce a noise prior to constraining the noise pattern from a practical view. Consequently, our proposed method realizes to automatically and simultaneously model the noise and estimate FBN under a unified framework. In summary, the contributions of our proposed method are highlighted as follows:

1. Our method combines the noise depression and FBN estimation into a unified framework, making it capable of obtaining a clearer FBN estimation and higher accuracy on disease diagnosis.
2. The modification of the data-fitting term can be interpreted as a traditional fitting term on a transformed data series, making it possible to modify a series of traditional methods to support noise modeling. Meanwhile, such modifications can make good use of existing optimization methods, which are both cheap and convenient.
3. The noise pattern can be fitted with the help of extra prior knowledge, which can be independently adjusted to adapt to the current task.
4. The earlier two points jointly constitute a plug-and-play noise module capable of being embedded into different methods and adjusted according to different noise priors.

## 2. Materials and methods

### 2.1. Data acquisition and preprocessing

For the participants in this study, we simply utilize a well-known and publicly available dataset, that is, Autism Brain Imaging Data Exchange (ABIDE) as in several recent studies (16, 26). The autism criteria sets in the Diagnostic and Statistical Manual of Mental Disorders, 4th Edition, Text Revision (DSM-IV-TR) (27) are adopted to diagnose ASD from NC. Above all, 45 ASD subjects (36 males and nine females) and 47 NC subjects (36 males and 11 females) between 7 and 15 year of age are included, with gender, age, and full intelligence quotient (FIQ) not differing significantly from ASD to NC. The detailed demographic information of the participant is given in Table 1.

**Table 1:** Demographic information of the subjects.

	**ASD** **(*N* = 45)**	**NC** **(*N* = 47)**	***p*-value**
FIQ (mean ± SD)	106.8 ± 17.4	113.3 ± 14.1	0.0510
Age (year ± SD)	11.1 ± 2.3	11.1 ± 2.3	0.7773^†^
Gender (M/F)	36/9	36/11	0.2135^*^
ADOS (mean ± SD)	13.7 ± 5.0	-	-
ADI-R (mean ± SD)	32.2 ± 14.3^‡^	-	-

FIQ, full intelligence quotient; ADOS, Autism Diagnostic Observation Schedule; ADI-R, Autism Diagnostic Interview-Revised.

* The *p*-value was obtained by the chi-squared test.

† The *p*-value was obtained by a two-sample two-tailed *t*-test.

‡ Two patients do not have the ADI-R score.

The resting-state fMRI scanning of all subjects is conducted using a 3T Siemens Allegra scanner within 6 min. During the scanning procedure, all subjects were asked to relax with their eyes open and focus on a white fixed cross in the middle of a black background projected on a screen. These requirements ensure the subjects to focus their attention and prevent meditation with the eyes closed, thereby avoiding violent neural activity. The parameters for acquiring images include: flip angle = 90°, 180 volumes per scan, 33 slices per volume, TR/TE = 2, 000/15 *ms*, and 4.0-*mm* voxel thickness (28).

After data acquisition, we conduct the statistical parametric mapping (SPM8) (http://www.fil.ion.ucl.ac.uk/spm/software/spm8/) as the preprocessing toolbox to preprocess the fMRI data. In particular, we remove the first 10 RS-fMRI images of each subject. The remaining images were spatially normalized into the Montreal Neurological Institute (MNI) template space with the resolution 3 × 3 × 3 *mm*. Then, the regression of nuisance signals (ventricle, white matter, global signals, and head motion with Friston 24-parameter model), signal detrending, and band-pass filtering (0.01–0.08 Hz) (29–31) are included for further corrections. After that, each preprocessed image was parcellated into 116 ROIs according to the automated anatomical labeling (AAL) atlas (32). Finally, these time series were as the data matrix, **X** ∈ ℝ^170×116^, where 170 denotes the total number of temporal image volumes and 116 denotes the total number of ROIs. In our study, we focus on the first 90 ROIs that belong to the cerebrum as our regions of interest, which are utilized in most studies using AAL. Thus, the data matrix size is reformed as **X** ∈ ℝ^170×90^.

### 2.2. Related methods

After data preparation, the next task is the FBN construction. In this study, we briefly review two FBN estimation approaches, that is, PC (33) and SR (22), which are all closely related to this study.

The notations used in the rest article are presented beforehand as follows. Bold uppercase letters are used to denote matrices, bold lowercase letters are used to denote vectors, and normal italic letters to denote scalars. The *i*th column of matrix **X** is denoted as **x**_*i*_, and the element of **X** at *i*th row and *j*th column is denoted as *x*_*ij*_. ∥ · ∥_*F*_, ∥ · ∥_2_, and ∥ · ∥_1_ denote the Frobenius norm, L2-norm, and L1-norm, respectively. | · | denotes the determinant of a matrix or the absolute value of a scalar. We further denote the transpose operator, the trace operator, and the inverse of a matrix **X** as **X**^*T*^, tr(**X**), and **X**^−1^.

#### 2.2.1. Pearson's correlation

The Pearson's correlation is the simplest and most commonly used method for estimating FBNs (33). **X** ∈ ℝ^*T*×*N*^ denote the fMRI data matrix (i.e., the BOLD signals), where *T* and *N* denote the number of time points in each series and the number of ROIs, respectively. ${\text{x}}_{i}\in {\mathbb{R}}^{T}\left(i=1,\cdots \;,N\right)$ denote the time series of the *i*th ROI, and then we can calculate the weight *w*_*ij*_ of the network connection between the *i*th and *j*th ROIs by PC as follows:


(1) $$\begin{array}{l} w_{ij}=\frac{{\left({\text{x}}_{i}-{\overset{-}{\text{x}}}_{i}\right)}^{T}\left({\text{x}}_{j}-{\overset{-}{\text{x}}}_{j}\right)}{\sqrt{{\left({\text{x}}_{i}-{\overset{-}{\text{x}}}_{i}\right)}^{T}\left({\text{x}}_{i}-{\overset{-}{\text{x}}}_{i}\right)}\sqrt{{\left({\text{x}}_{j}-{\overset{-}{\text{x}}}_{j}\right)}^{T}\left({\text{x}}_{j}-{\overset{-}{\text{x}}}_{j}\right)}}, \end{array}$$

where ${\overset{-}{\text{x}}}_{i}\in {\mathbb{R}}^{T}$ denotes a mean vector with all entries being the mean value of all elements in **x**_*i*_.

Without the loss of generality, suppose that the fMRI data have been centralized and normalized by ${\text{x}}_{i}=\left({\text{x}}_{i}-{\overset{-}{\text{x}}}_{i}\right)/\sqrt{{\left({\text{x}}_{i}-{\overset{-}{\text{x}}}_{i}\right)}^{T}\left({\text{x}}_{i}-{\overset{-}{\text{x}}}_{i}\right)}$, the weight can be simplified as the form $w_{ij}={\text{x}}_{i}^{T}{\text{x}}_{j}$, which corresponds to the optimal solution of the following problem:


(2) $$\begin{array}{l} \underset{\text{W}}{\min}∥\text{W}-{\text{X}}^{T}\text{X}{∥}_{F}^{2}, \end{array}$$

where $\text{W}=\left(w_{ij}\right)\in {\mathbb{R}}^{N\times N}$ is the edge weight matrix of the estimated FBN. The above remodels PC in a perspective of optimization and benefits to develop new flexible FBN estimation methods based on PC (16).

In general, PC-based estimation methods produce a dense FBN, in which the ROIs are fully connected, with some connections being noisy or uninformative. In order to filter out such connections, thresholding or sparsity-induced constraint is generally used to sparsify the estimated FBN. For more details of the thresholding scheme, see Fornito et al. (34).

#### 2.2.2. Sparse representation

Although PC is simple and empirically effective in building FBN, it can only measure the full correlation and neglect the interaction among multiple ROIs. Instead of measuring full correlation, PC-based methods aim to estimate more reliable connections between two ROIs by regressing out the confounding effect from other ROIs (22). Nevertheless, such an approach might be ill-posed due to the inverse calculation of a singular sample covariance matrix. To address this issue, an L1-norm regularizer is incorporated into the partial correlation model, resulting in the SR-based FBN estimation (22) as follows:


(3) minwij∑i=1N∥xi-∑j=1j≠iNwijxj∥22+λ1∑j=1j≠iN|wij|

which can be further rewritten as the equivalent matrix form:


(4) $$\begin{array}{l} \underset{\text{W}}{\min}\;∥\text{X}-\text{X}\text{W}{∥}_{F}^{2}+\lambda_{1}∥\text{W}{∥}_{1}\\ \;s.t.\;w_{ii}=0,\forall i=1,\cdots \;,N \end{array}$$

where constraint *w*_*ii*_ = 0 is used to remove **x**_*i*_ from **X** to avoid the trivial solution, λ_1_ is a regularized parameter that controls the sparsity of the estimated FBN and benefits for achieving a stable solution (35).

### 2.3. Proposed methods

As two typical examples, PC and SR have been demonstrated to be more sensitive than some complex higher-order methods (13). Nevertheless, since the original data points in the time series possibly contain “noise”, the estimated FBNs are often heavily influenced by the quality of the observed data and result in the representation noise of the network connections (25). To address this issue, in this section, we mainly focus on the baseline method SR and introduce a noise modeling scheme for FBN estimation.

#### 2.3.1. SRAND: Sparse representation with adaptive noise depression

Suppose **X** to be the noisy fMRI data (assumed to be centralized and normalized), then the first term in Equation (3) can be rewritten as follows:


(5) ∑i=1N∥xi-∑j=1j≠iNwijxj∥22=∑i=1N∥(xiclear+ni)-∑j=1j≠iNwij(xjclear+nj)∥22=∑i=1N∥(xiclear-∑j=1j≠iNwijxjclear)+(ni-∑j=1j≠iNwijnj)∥22

where the superscript *clear* denotes the clean data and **n**_*i*_ denotes the noise term of the *i*th ROI.

We can see that the total representation error in the objective function can be rewritten by the sum of the representation error on clean fMRI data and that caused by the noise of the fMRI data. However, traditional SR takes no account of the influence brought by such noise-caused error, and simply assumes that the representation error terms are identically independently distributed [i.i.d., equivalent to the L2-norm in Equation (3)]. To this end, we introduce to model of the error to depress the noise influence on FBN estimation. Specifically, we consider measuring the partial correlation and assuming the representation error between the *i*th ROI and the *j*th ROI follows Gaussian distribution with a non-diagonal precision matrix, that is,


(6) xi~N(xi|∑j=1j≠iNxjwij,Ω-1),

where **Ω** denotes the precision matrix (i.e., the inverse covariance matrix). Here, non-diagonal condition on **Ω** indicates the noise term to be non-i.i.d, which is more practical to measure the dependent relationship between noises among time series. Moreover, we assume that the precision matrix is identical for noise terms between different ROIs so that to capture the noise pattern implied in time series rather than ROIs.

Taking the negative logarithm of Equation (6), we obtain the maximum-likelihood estimation (MLE) of *w*_*ij*_ and **Ω** by minimizing


(7) minwij,Ω (xi-∑j=1j≠iNxjwij)TΩ(xi-∑j=1j≠iNxjwij)-ln|Ω|,

In subsequent, we consider the optimization problem corresponding to *w*_*ij*_ by fixing **Ω**. We define a new transformed fMRI time series ${\text{y}}_{i}=\Omega^{\frac{1}{2}}{\text{x}}_{i}$, then the objective term related to *w*_*ij*_ in the Equation (7) can be rewritten as follows:


(8) (xi-∑j=1j≠iNxjwij)TΩ(xi-∑j=1j≠iNxjwij)=∥yi-∑j=1j≠iNyjwij∥22.

Consequently, the optimization problem of *w*_*ij*_ embedded with the newly added noise depression module can be formulated as follows:


(9) minwij∑i=1N∥yi-∑j=1j≠iNyjwij∥22+λ1∑j=1j≠iN|wij|

or equivalently as its matrix form


(10) $$\begin{array}{l} \underset{\text{W}}{\min}\;∥\text{Y}-\text{Y}\text{W}{∥}_{F}^{2}+\lambda_{1}∥\text{W}{∥}_{1}.\\ \;s.t.\;w_{ii}=0,\forall i=1,\cdots \;,N \end{array}$$

We can see that the above optimization problem coincides with the traditional SR on the transformed fMRI data series **y**_*i*_, which can be easily solved by existing SR-based optimization methods (22). In contrast, the coincidence promotes us to embed such noise modeling into other FBN estimation methods following a similar pipeline. In other words, such transformation on fMRI data can be applied as a plug-and-play noise module to elegantly modify the traditional methods, making them capable of depressing noise.

#### 2.3.2. Adaptive noise modeling with different priors

After the optimization of the edge weights *w*_*ij*_, we turn to the noise modeling term **Ω**. We first reformulate Equation (7) into matrix form about **Ω** as follows:


(11) $$\begin{array}{l} \underset{\Omega }{\min}\text{ tr}\left[\Omega \left(\text{X}-\text{X}\text{W}\right){\left(\text{X}-\text{X}\text{W}\right)}^{T}\right]-N\ln|\Omega | \end{array}$$

The element at the *i*th row and *j*th column of **Ω** measures the noise relationship between the *i*th and *j*th time points. If the value approaches to zero, then the two time points are conditionally independent and vice versa. Such modeling can capture the noise dependence among time series, which is more practical than i.i.d. assumption.

However, similar to the estimation of FBN, due to the computation of **Ω** involving inversing the covariance matrix, directly optimizing **Ω** based on objective function (12) is often ill-posed. Thus, it makes sense to impose a prior to constrain the structure of **Ω**. We embed such prior *via* a regularizer on **Ω** and present the following universal form for optimizing **Ω**,


(12) $$\begin{array}{l} \underset{\Omega }{\min}\text{ tr}\left[\Omega \left(\text{X}-\text{X}\text{W}\right){\left(\text{X}-\text{X}\text{W}\right)}^{T}\right]-N\ln|\Omega |+\lambda_{2}R\left(\Omega \right), \end{array}$$

where *R*(**Ω**) is a regularized term, λ_2_ is a trade-off parameter.

Considering that only the noises which change with time series regularly appear to be correlated in **Ω**, while irregular noises among time series are usually independent of each other, it is natural to impose an L1-norm penalty to model such sparse structure, resulting in the following:


(13) $$\begin{array}{l} \underset{\Omega }{\min}\text{ tr}\left[\Omega \left(\text{X}-\text{X}\text{W}\right){\left(\text{X}-\text{X}\text{W}\right)}^{T}\right]-N\ln|\Omega |+\lambda_{2}∥\Omega {∥}_{1}, \end{array}$$

which can be solved by existing optimization methods, for example, the method of Meinshausen and Bühlmann (36) or the classical graphical lasso (37).

By combining the objective functions and joining all constraints in Equations (10) and (13), the sparse representation based on adaptive noise depression (SRAND) with L1-norm constraint can be summarized as follows:


(14) $$\begin{array}{l} \underset{\text{W},\Omega }{\min}\text{ tr}\left[{\left(\text{X}-\text{X}\text{W}\right)}^{T}\Omega \left(\text{X}-\text{X}\text{W}\right)\right]\\ \;-N\ln|\Omega |+\lambda_{1}∥\text{W}{∥}_{1}+\lambda_{2}∥\Omega {∥}_{1}\\ \;s.t.\;w_{ii}=0,\forall i=1,\cdots \;,N. \end{array}$$

Consequently, the procedure of SRAND with L1-norm is listed in Algorithm 1.

**Algorithm 1:** SRAND with L1-norm.

**Input:** Data matrix **X**, parameters λ_1_ and λ_2_
**Output:** Constructed FBN **W**
Initialize **Ω** = **I**
**while** not converge **do**
Update **W** by solving the problem in Equation (10)
Update **Ω** by solving the problem in Equation (13)
**end while**

In addition to the above kind of prior that directly imposes specific structure on the noise pattern, in the following, we introduce another prior, Wishart distribution as the prior of **Ω** to embed structure implicitly. In particular, the Wishart distribution is given by the following:


(15) $$\begin{array}{l} W\left(\Omega |\Sigma ,\nu \right)=B\left(\Sigma ,\nu \right)|\Omega {|}^{\left(\nu -T-1\right)/2}\exp\left(-\frac{1}{2}\text{tr}\left(\Sigma^{-1}\Omega \right)\right), \end{array}$$

where


$$\begin{array}{l} B\left(\Sigma ,\nu \right)=|\Sigma {|}^{-\nu /2}{\left(2^{\nu T/2}\pi^{T\left(T-1\right)/4}\overset{T}{\underset{i=1}{\prod }}\Gamma \left(\frac{\nu +1-i}{2}\right)\right)}^{-1} \end{array}$$

Is a scaler irrelevant to **Ω**, ν is the *number of degrees of freedom* and restricted to ν > *T* − 1, **Σ** is a *N* × *N* symmetric, positive definite matrix.

Then we assume that the precision matrix **Ω** is subject to Wishart distribution,


(16) $$\begin{array}{l} \Omega \sim W\left(\Omega |\Sigma ,\nu \right). \end{array}$$

By combining Equations (6) and (15), we obtain the conditional distribution of **Ω** satisfying,


(17) $$\begin{array}{l} p\left(\Omega |\Sigma ,\nu ,\text{W}\right)\\ \propto \overset{N}{\underset{i=1}{\prod }}p\left({\text{x}}_{i}|\text{X},{\text{w}}_{i},\Omega \right)p\left(\Omega |\Sigma ,\nu \right)\\ \propto \overset{N}{\underset{i=1}{\prod }}|\Omega {|}^{1/2}\exp\left(-\frac{1}{2}\text{tr}\left(\left({\text{x}}_{i}-\text{X}{\text{w}}_{i}\right){\left({\text{x}}_{i}-\text{X}{\text{w}}_{i}\right)}^{T}\Omega \right)\right)\\ \times |\Omega {|}^{\left(\nu -T-1\right)/2}\exp\left(-\frac{1}{2}\text{tr}\left(\Sigma^{-1}\Omega \right)\right), \end{array}$$

where **w**_*i*_ denotes the *i*th column of **W** and the terms only relevant to the optimization of *w*_*ij*_ is omitted. From Equation (17), we see, Wishart prior is conjugate to the precision matrix **Ω** of the multi-variate Gaussian distribution.

In conclusion, we reform Equation (17) and obtain the variational posterior *q*(**Ω**) *via* approximation inference (38) that follows:


(18) $$\begin{array}{l} q\left(\Omega \right)\propto |\Omega {|}^{\left(N+\nu -T-1\right)/2}\exp\\ \times \left(-\frac{1}{2}\text{tr}\left(\left(\left(\text{X}-\text{X}\text{W}\right){\left(\text{X}-\text{X}\text{W}\right)}^{T}+\Sigma^{-1}\right)\Omega \right)\right). \end{array}$$

Then, the optimal solution of **Ω** can be computed analytically as follows:


(19) $$\begin{array}{l} \Omega =\left(N+\nu \right){\left(\left(\text{X}-\text{X}\text{W}\right){\left(\text{X}-\text{X}\text{W}\right)}^{T}+\Sigma^{-1}\right)}^{-1}, \end{array}$$

which is equivalent to the optimal solution of the following optimization problem,


(20) $$\begin{array}{l} \underset{\Omega }{\min}\text{ tr}\left[\Omega \left(\left(\text{X}-\text{X}\text{W}\right){\left(\text{X}-\text{X}\text{W}\right)}^{T}+\Sigma^{-1}\right)\right]-\left(N+\nu \right)\ln|\Omega |. \end{array}$$

According to the form of the optimal solution, the structure of **Ω** is partially governed by the structure of the prior matrix **Σ**; thus it is natural to embed prior structure *via* **Σ** implicitly. Without the loss of generality, in this article, we consider **Σ** = α^−1^**I** as an infinitely broad prior, so that the structure of **Ω** is almost learned from the data entirely.

To sum up, the overall objective function joining all constraints in Equations (10) and (20), the SRAND with Wishart prior constraint can be summarized as follows:


(21) $$\begin{array}{l} \underset{\text{W},\Omega }{\min}\text{ tr}\left[\Omega \left(\left(\text{X}-\text{X}\text{W}\right){\left(\text{X}-\text{X}\text{W}\right)}^{T}+\Sigma^{-1}\right)\right]\\ \;-\left(N+\nu \right)\ln|\Omega |+\lambda_{1}∥\text{W}{∥}_{1}\\ \;s.t.\;w_{ii}=0,\forall i=1,\cdots \;,N. \end{array}$$

Consequently, the procedure of SRAND with Wishart prior is listed in Algorithm 2.

**Algorithm 2:** SRAND with Wishart prior.

**Input:** Data matrix **X**, parameters λ_1_, ν, **Σ**
**Output:** Constructed FBN **W**
Initialize **Ω** = **I**
**while** not converge **do**
Update **W** by solving the problem in Equation (10)
Update **Ω** by Equation (19)
**end while**

## 3. Experiments and results

### 3.1. Experimental setting

#### 3.1.1. FBN construction

In this section, we estimate FBNs on ABIDE database using different methods, including SR and the proposed SRAND with Wishart prior. In addition, we also conducted experiments using PC and PCAND (i.e., PC modified *via* adaptive noise depression) with Wishart prior. In this study, we fix the Wishart distribution with equal diagonal entries as the prior to gain an infinitely broad prior and to evaluate the influence of the same prior on different comparison methods. The comparison between different priors on the same method is followed in the subsequent section. In general, each SR-based method contains one or more hyperparameters for regularization, which may significantly influence the network structure and then the ultimate classification results (39). Therefore, for each regularized parameter, we build multiple FBNs on different parameter values in the candidate range [0.05, 0.1, ⋯ , 0.95, 1] and then search the optimal parameter value *via* a separate parameter selection procedure.

#### 3.1.2. Feature selection and classification

After obtaining the estimated FBNs, we subsequently utilize them to identify participants with ASD from NCs. In our experiments, the upper triangular edge weights of the FBNs are selected as the input features since the FBN matrix is symmetric. In particular, 90 nodes (i.e., number of ROIs) produce 90 × (90 − 1)/2 = 4, 005 dimensions of the feature. Compared with the small sample size of 92, it is still too high to ensure the good generalization ability of the classifier, which obviously affects the final classification accuracy. To address this problem, we adopt the simplest *t*-test with *p* = 0.01 as feature selection method. After selection of the most-relevant features, we use the most popular support vector machine (SVM) (linear kernel with default parameter *C* = 1) as our classifier for disease identification (40).

Furthermore, we test the involved FBN estimation methods by the leave-one-out cross-validation (LOOCV), in which experiments repeat for *K* times (i.e., the total number of subjects) for each subject as the testing set, while the rest subjects are used as the training set to select features and train classifier. Moreover, in order to determine the optimal value of the regularization parameter, an inner LOOCV is conducted on the training data *via* a grid search on the candidate range of parameters, which is based on the metric of classification accuracy.

**Figure 1:**
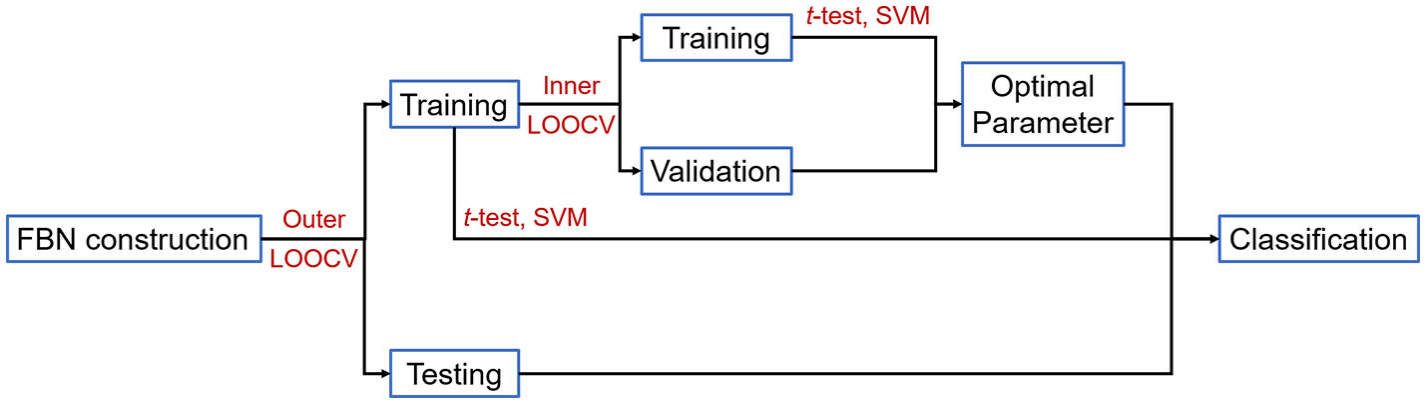
Experiment procedure based on the estimated FBNs.

The overall detailed pipeline of our experiments is shown in Figure 1.

### 3.2. Results

#### 3.2.1. FBN visualization

**Figure 2:**
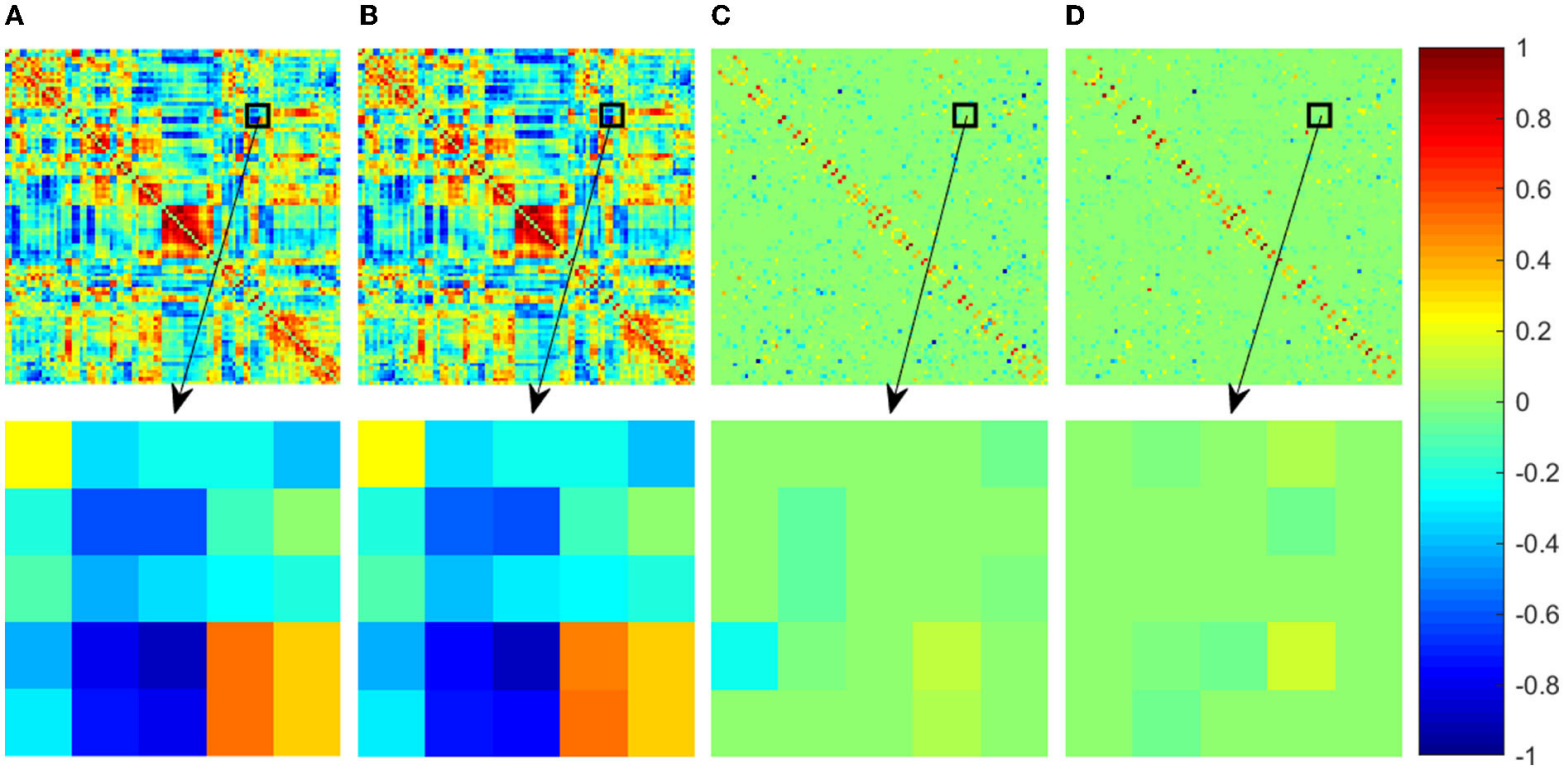
Adjacency matrices of the FBNs estimated by four comparison methods. The first row shows the whole adjacency matrix and the second row shows the enlarged part corresponding to the black box in the first row. For a convenient comparison between the visualized results, the elements in the adjacency matrices have been normalized into the interval [−1, 1]. **(A)** PC, **(B)** PCAND, **(C)** SR, and **(D)** SRAND.

In order to compare the results of different FBN estimation methods intuitively, we first take one subject from ABIDE dataset as an example to visualize the adjacency matrices of the FBNs estimated by the four comparison methods in Figure 1. Since the FBN is generally dense for PC-based methods, in order to show the changes more clearly, we enlarge part of the FBNs (indicated by the black box in Figure 2) and show the enlarged part (the second row in Figure 2).

It can be observed that the PC-based FBN is significantly different from those estimated by SR-based methods since they use different data-fitting terms to capture full correlation and partial correlation between ROIs, respectively. In contrast, the FBNs constructed based on the same data-fidelity term share similar topological structure, for example, PCAND is similar to that of PC and SRAND is similar to that of SR. Moreover, compared with PC and SR, the FBN estimated by PCAND and SRAND can further weaken or remove the noisy or weak connections and produce an even clearer topological structure. Specifically, although there is no sparsity constraint on estimating FBN by PCAND, it can weaken some of the dense connections constructed by the original PC (as shown by the brighter color blocks that indicate lower connection weights). As for SRAND, it can further remove the weak connections on the basis of the sparsity-contrained SR.

#### 3.2.2. ASD identification

In this study, we adopt three quantitative metrics, including accuracy (ACC), sensitivity or true positive rate (SEN) and specificity or true negative rate (SPE) to evaluate the classfication performance of different methods. The mathematical definitions of the first three measures are given as follows:


(22) $$\begin{array}{l} ACC=\frac{TP+TN}{TP+TN+FP+FN}\\ SEN=\frac{TP}{TP+FN}\\ SPE=\frac{TN}{TN+FP}. \end{array}$$

where TP, TN, FP, and FN indicate true positive, true negative, false positive, and false negative, respectively. It should be noted that in this study, we treat the subjects with ASD as the positive class, while the NCs as the negative class.

The ASD vs. NC classification results on ABIDE dataset are reported in Table 2. As can be seen, the PC-based methods perform better than SR-based ones in our experiment. A similar problem has also been revealed in other studies, which can be blamed on the ill-posed computation of inverse covariance matrix with high feature dimension (16). Nevertheless, both modified methods enjoy better performance than the original ones, increasing classification accuracy by approximately 3.26 and 13.04%, respectively. The aforementioned results can be attributed to the adaptive noise depression module that can further filter out the noisy or weak connections in the estimated FBN and provide clearer connections highly related to neural disorders.

**Table 2:** Classification performance corresponding to different FBN estimation methods on ABIDE dataset.

**Method**	**Accuracy**	**Sensitivity**	**Specificity**
PC	0.6848	0.7333	0.6383
SR	0.5435	0.6000	0.4894
PCAND	**0.7174**	**0.7333**	0.7021
SRAND	0.6739	0.5556	**0.7872**

The bold values mean the best results corresponding to the performance metrics.

## 4. Discussion

### 4.1. Sensitivity to network model parameters

**Figure 3:**
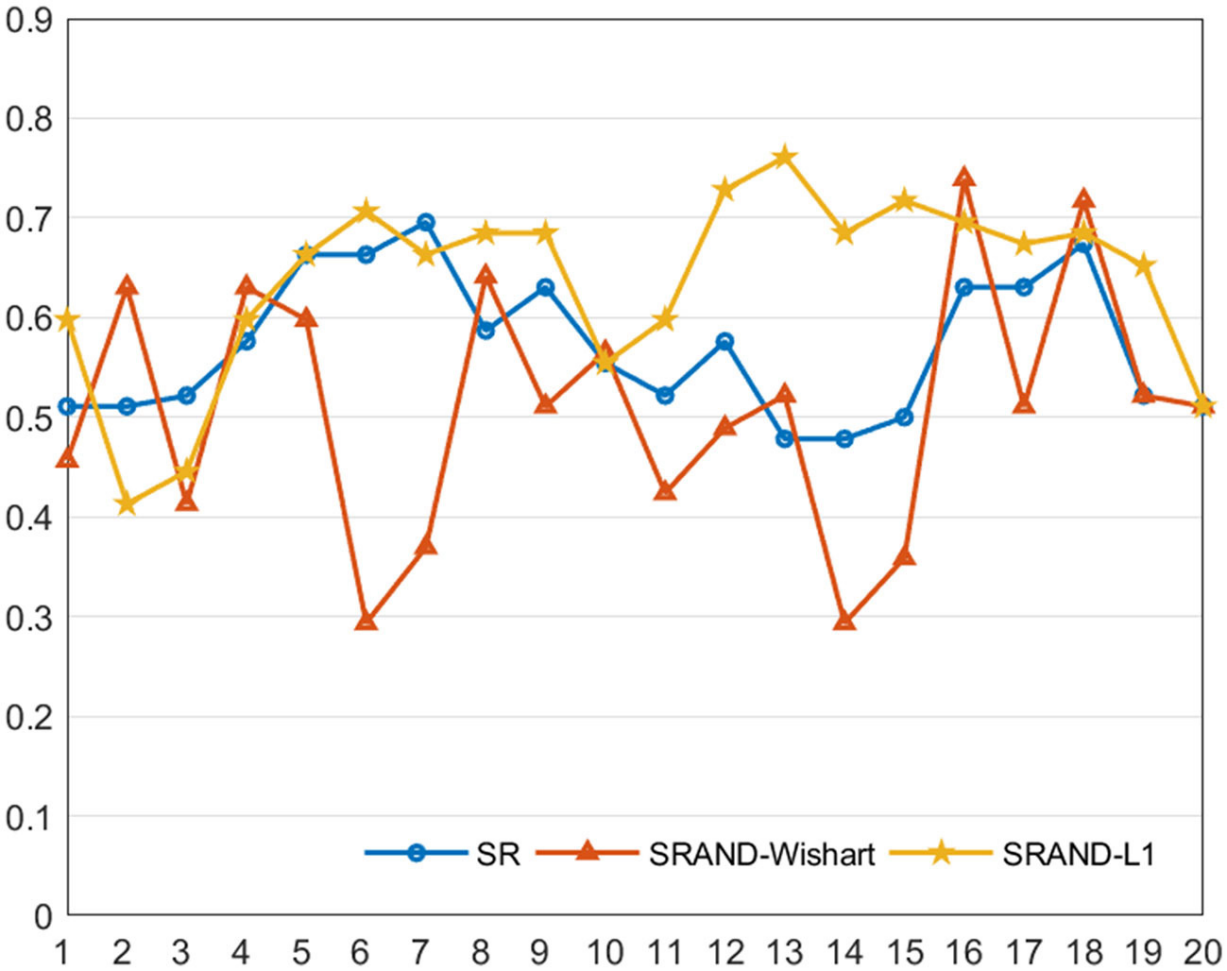
Classification accuracy of the FBN estimated by three comparison methods on 20 regularized parameters.

In general, the trade-off parameters in the FBN estimation methods play an important role to influence the ultimate classification performance (17, 41). To investigate the sensitivity of the proposed method to different parameter values, we repeat ASD classification experiments based on different parameter combinations and compute the classification accuracy *via* LOOCV on all of the subjects. In addition to SRAND with Wishart prior, in this experiment, we also include the version with sparsity prior as compared to evaluate the influence of different priors. For convenient, we denote the SRAND with Wishart prior and sparsity prior as SRAND-Wishart, and SRAND-L1, separately. In order to compare the sensitivity of the three methods, SR, SRAND-Wishart and SRAND-L1 simultaneously and conveniently, we fix the second parameter of SRAND-L1 as λ_2_ = 0.1, so that the three methods have the same comparable parameter. Such operation is relatively fair for SR and SRAND-Wishart. Indeed, the fixed second parameter limits the freedom of the model and may keep SRAND-L1 away from the potential higher accuracy. The results are reported in Figure 3.

We note that most of the methods are sensitive to the parameters. Compared with the traditional SR-based methods, SRAND-Wishart is highly affected by varied parameters and even gains lower accuracy than the traditional ones. Such results are reasonable since Wishart prior actually imposes no structure constraint on the noise distribution, and the computation of **Ω** is determined by the fMRI data. Thus, computing **Ω** is generally ill-posed and sensitive to the parameter due to the limited sample size. In contrast, SRAND-L1 is less likely affected by parameters and thus achieves more stable results. In addition, SRAND-L1 achieves an improvement of the classification performance on most of the parameter values. Such results can be attributed to the well chosen prior that fits the noise structure. Therefore, we believe that appropriate adaptive noise depression module could eliminate bad effects on the FBN estimation and benefit the ultimate disease diagnosis.

**Figure 4:**
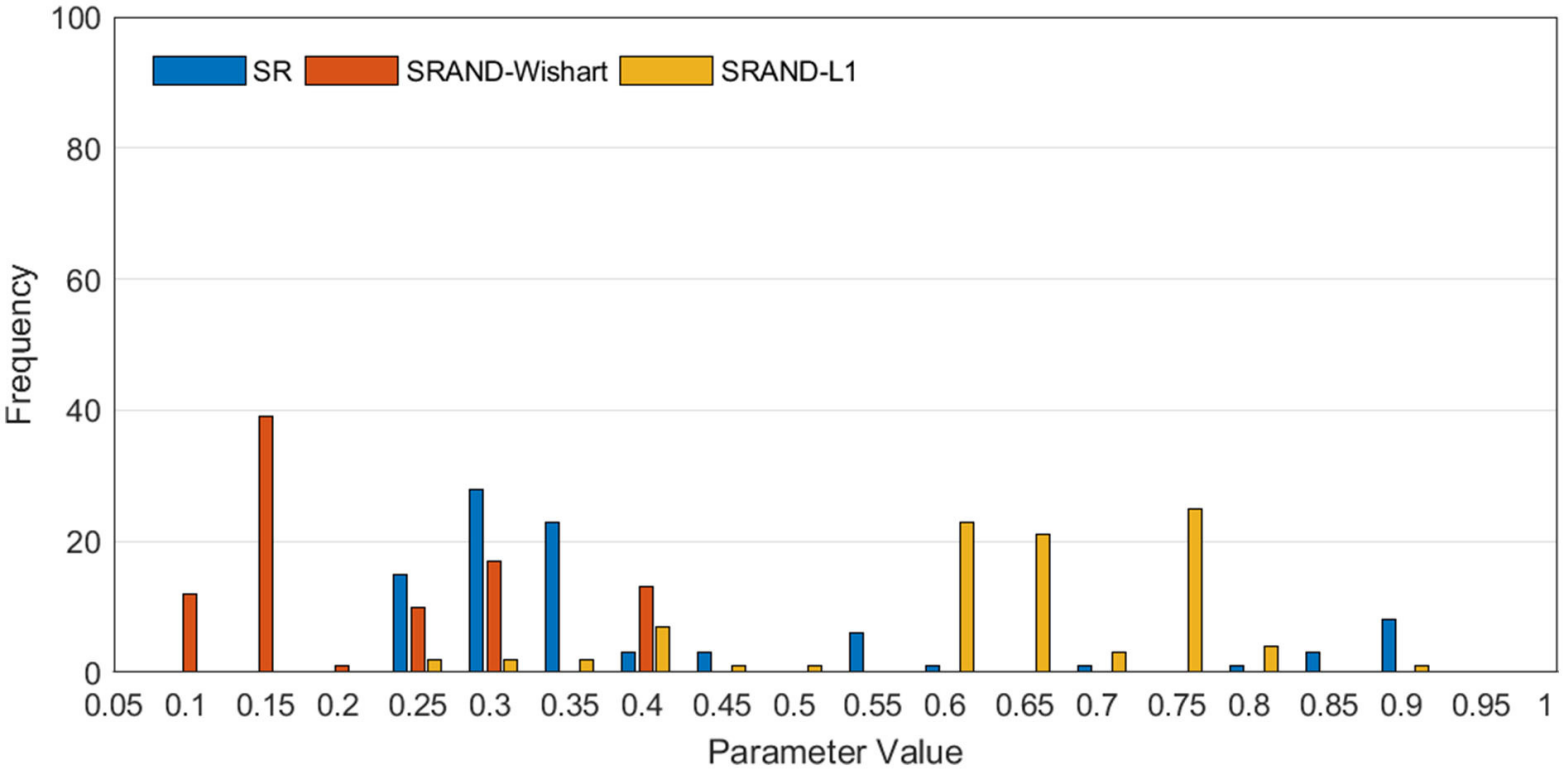
Frequency of the selected optimal values of parameter λ in the inner loops, where the horizontal axis represents the candidate parameter values, and the vertical axis represents the frequency of the parameter value being selected *via* parameter selection.

In addition to the comparison between classification accuracies on different parameters, we also present the value distribution of the optimal parameters selected in the inner loops, as shown in Figure 4. We can find that the optimal parameters of all SR-based methods concentrate around some fixed λ, that is, λ = 0.3 for SR, λ = 0.15 for SRAND-Wishart, and λ = 0.65 for SRAND-L1. Such concentricity also implies the sensitivity of all SR-based methods that they prefer some fixed parameter value than the scattered ones.

### 4.2. Discriminative features

**Figure 5:**
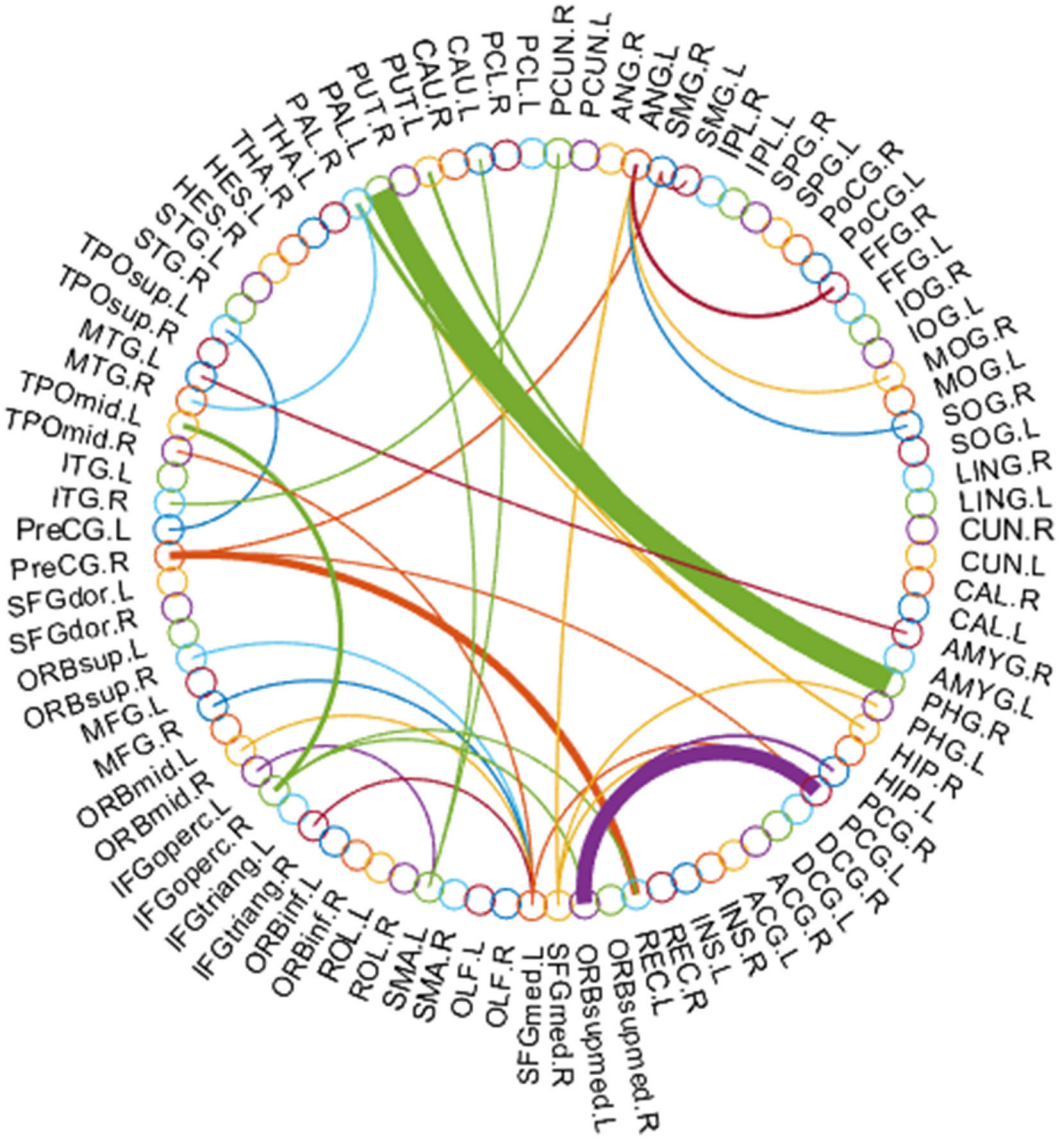
Most discriminative connections between ASD and NC for the first 90 ROIs of the AAL template.

In this subsection, we use the estimated FBN of PCAND as an example to explore which features contribute the most to ASD identification in our experiments. Specifically, we apply *t*-test with a *p*-value of 0.001 to select discriminative features based on the FBN constructed by PCAND. The top 66 most discriminative connections are visualized based on the first 90 ROIs of AAL template (32) in Figure 5, where the thickness of the arc shows the discriminative power. From Figure 5, we can find that the most discriminative features focus on the brain regions, including frontal, parahippocampus and pallidum, and so on. The frontal lobe is key to communication and cognitive function and is known to be implicated in ASD (42). Parahippocampus has been shown to have hypoactivation in scene recognition, in line with the notion of peaks and valleys of neural recruitment in individuals with ASD (43). Pallidum enlargement has been found in ASD compared with NC and may be a possible related factor in stereotypic behavior and social bonding (44). Similar findings have also been presented in previous studies (45–47).

## 5. Conclusion

Sparse representation is one of the most commonly used schemes for estimating FBNs due to its simplicity and relatively clearer network connections. Nevertheless, the SR scheme is still trapped in noisy or weak connections due to the noise introduced *via* data acquisition. In this study, we embed a noise module based on which the prior of noise pattern can be naturally incorporated in the form of regularizers. Such module has been illustrated to be plug and play that is capable of being embedded into different methods and adjusted according to different noise priors. To evaluate the effectiveness of the proposed scheme, we conduct experiments on the ABIDE database to identify subjects with ASD from normal controls. The experimental results demonstrate that the proposed method can achieve better performance than the baseline method.

## Data availability statement

The original contributions presented in the study are included in the article/supplementary material, further inquiries can be directed to the corresponding author.

## Ethics statement

Ethical review and approval/written informed consent to participate was not required in accordance with local legislation and institutional guidelines.

## Author contributions

DM and LP designed the study and drafted the manuscript. LP collected the MRI data. LP and XG analyzed and interpreted the results of the data. XG and DM revised the manuscript. All authors approved the final manuscript.

## References

[1] Wee CY Yap PT Shen D. Diagnosis of autism spectrum disorders using temporally distinct resting-state functional connectivity networks. CNS Neurosci Therapeut. (2016) 22:212–9. 10.1111/cns.12499 26821773 PMC4839002

[2] Dadi K Rahim M Abraham A Chyzhyk D Milham M Thirion B. Benchmarking functional connectome-based predictive models for resting-state fMRI. Neuroimage. (2019) 192:115–34. 10.1016/j.neuroimage.2019.02.062 30836146

[3] Maenner MJ Shaw KA Bakian AV Bilder DA Durkin MS Esler A. Prevalence and characteristics of autism spectrum disorder among children aged 8 years autism and developmental disabilities monitoring network, 11 sites, United States, 2018. MMWR Surveill Summ. (2021) 70:1. 10.15585/mmwr.ss7011a1 34855725 PMC8639024

[4] Guthrie W Swineford LB Nottke C Wetherby AM. Early diagnosis of autism spectrum disorder: stability and change in clinical diagnosis and symptom presentation. J Child Psychol Psychiatry. (2013) 54:582–90. 10.1111/jcpp.12008 23078094 PMC3556369

[5] O' Roak BJ Vives L Fu W Egertson JD Stanaway IB Phelps IG. Multiplex targeted sequencing identifies recurrently mutated genes in autism spectrum disorders. Science. (2012) 338:1619–22. 10.1126/science.1227764 23160955 PMC3528801

[6] Zhang Y Li N Li C Zhang Z Teng H Wang Y. Genetic evidence of gender difference in autism spectrum disorder supports the female-protective effect. Transl Psychiatry. (2020) 10:1–10. 10.1038/s41398-020-0699-8 32066658 PMC7026157

[7] Wass S. Distortions and disconnections: disrupted brain connectivity in autism. Brain Cogn. (2011) 75:18–28. 10.1016/j.bandc.2010.10.005 21055864

[8] Drysdale AT Grosenick L Downar J Dunlop K Mansouri F Meng Y. Resting-state connectivity biomarkers define neurophysiological subtypes of depression. Nat Med. (2017) 23:28–38. 10.1038/nm.4246 27918562 PMC5624035

[9] Abraham A Milham MP Di Martino A Craddock RC Samaras D Thirion B. Deriving reproducible biomarkers from multi-site resting-state data: an Autism-based example. Neuroimage. (2017) 147:736–45. 10.1016/j.neuroimage.2016.10.045 27865923

[10] Fox MD Raichle ME. Spontaneous fluctuations in brain activity observed with functional magnetic resonance imaging. Nat Rev Neurosci. (2007) 8:700–11. 10.1038/nrn2201 17704812

[11] Starck T Nikkinen J Rahko J Remes J Hurtig T Haapsamo H. Resting state fMRI reveals a default mode dissociation between retrosplenial and medial prefrontal subnetworks in ASD despite motion scrubbing. Front Hum Neurosci. (2013) 7:802. 10.3389/fnhum.2013.00802 24319422 PMC3837226

[12] Eslami T Mirjalili V Fong A Laird AR Saeed F. ASD-DiagNet: a hybrid learning approach for detection of autism spectrum disorder using fMRI data. Front Neuroinform. (2019) 13:70. 10.3389/fninf.2019.00070 31827430 PMC6890833

[13] Smith SM Miller KL Salimi-Khorshidi G Webster M Beckmann CF Nichols TE. Network modelling methods for FMRI. Neuroimage. (2011) 54:875–91. 10.1016/j.neuroimage.2010.08.063 20817103

[14] Maximo JO Cadena EJ Kana RK. The implications of brain connectivity in the neuropsychology of autism. Neuropsychol Rev. (2014) 24:16–31. 10.1007/s11065-014-9250-0 24496901 PMC4059500

[15] Hull JV Dokovna LB Jacokes ZJ Torgerson CM Irimia A Van Horn JD. Resting-state functional connectivity in autism spectrum disorders: a review. Front Psychiatry. (2017) 7:205. 10.3389/fpsyt.2016.00205 28101064 PMC5209637

[16] Li W Wang Z Zhang L Qiao L Shen D. Remodeling Pearson's correlation for functional brain network estimation and autism spectrum disorder identification. Front Neuroinform. (2017) 11:55. 10.3389/fninf.2017.00055 28912708 PMC5583214

[17] Xue Y Zhang L Qiao L Shen D. Estimating sparse functional brain networks with spatial constraints for MCI identification. PLoS One. (2020) 15:e0235039. 10.1371/journal.pone.0235039 32707574 PMC7381102

[18] Xu X Wang T Li W Li H Xu B Zhang M. Morphological, structural, and functional networks highlight the role of the cortical-subcortical circuit in individuals with subjective cognitive decline. Front Aging Neurosci. (2021) 13:394. 10.3389/fnagi.2021.688113 34305568 PMC8299728

[19] Khatri U Kwon GR. Alzheimer's disease diagnosis and biomarker analysis using resting-state functional MRI functional brain network with multi-measures features and hippocampal subfield and amygdala volume of structural MRI. Front Aging Neurosci. (2022) 14:818871. 10.3389/fnagi.2022.818871 35707703 PMC9190953

[20] Li WK Chen YC Xu XW Wang X Gao X. Human-Guided functional connectivity network estimation for chronic tinnitus identification: a modularity view. IEEE J Biomed Health Inform. (2022) 26:4849–58. 10.1109/JBHI.2022.3190277 35830394

[21] Li W Xu X Wang Z Peng L Wang P Gao X. Multiple connection pattern combination from single-mode data for mild cognitive impairment identification. Front Cell Dev Biol. (2021) 9:782727. 10.3389/fcell.2021.782727 34881247 PMC8645991

[22] Lee H Lee DS Kang H Kim BN Chung MK. Sparse brain network recovery under compressed sensing. IEEE Trans Med Imaging. (2011) 30:1154–1165. 10.1109/TMI.2011.2140380 21478072

[23] Huang S Li J Sun L Ye J Fleisher A Wu T. Learning brain connectivity of Alzheimer's disease by sparse inverse covariance estimation. Neuroimage. (2010) 50:935–49. 10.1016/j.neuroimage.2009.12.120 20079441 PMC3068623

[24] Poldrack RA Mumford JA Nichols TE. Handbook of functional MRI data analysis. New York, NY: Cambridge University Press.

[25] Li W Qiao L Zhang L Wang Z Shen D. Functional brain network estimation with time series self-scrubbing. IEEE J Biomed Health Inform. (2019) 23:2494–504. 10.1109/JBHI.2019.2893880 30668484 PMC6904893

[26] Di Martino A Yan CG Li Q Denio E Castellanos FX Alaerts K. The autism brain imaging data exchange: towards a large-scale evaluation of the intrinsic brain architecture in autism. Mol Psychiatry. (2014) 19:659–67. 10.1038/mp.2013.78 23774715 PMC4162310

[27] First MB France A Pincus HA. DSM-IV-TR guidebook. American Psychiatric Publishing, Inc.;. (2004).

[28] Zhao F Zhang X Thung KH Mao N Lee SW Shen D. Constructing multi-view high-order functional connectivity networks for diagnosis of autism spectrum disorder. IEEE Trans Biomed Eng. (2021) 69:1237–50. 10.1109/TBME.2021.3122813 34705632

[29] Satterthwaite TD Elliott MA Gerraty RT Ruparel K Loughead J Calkins ME. An improved framework for confound regression and filtering for control of motion artifact in the preprocessing of resting-state functional connectivity data. Neuroimage. (2013) 64:240–56. 10.1016/j.neuroimage.2012.08.052 22926292 PMC3811142

[30] Yan CG Cheung B Kelly C Colcombe S Craddock RC Di Martino A. A comprehensive assessment of regional variation in the impact of head micromovements on functional connectomics. Neuroimage. (2013) 76:183–201. 10.1016/j.neuroimage.2013.03.004 23499792 PMC3896129

[31] Xie Q Zhang X Rekik I Chen X Mao N Shen D. Constructing high-order functional connectivity network based on central moment features for diagnosis of autism spectrum disorder. PeerJ. (2021) 9:e11692. 10.7717/peerj.11692 34268010 PMC8269664

[32] Tzourio-Mazoyer N Landeau B Papathanassiou D Crivello F Etard O Delcroix N. Automated anatomical labeling of activations in SPM using a macroscopic anatomical parcellation of the MNI MRI single-subject brain. Neuroimage. (2002) 15:273–89. 10.1006/nimg.2001.0978 11771995

[33] Smith SM Vidaurre D Beckmann CF Glasser MF Jenkinson M Miller KL. Functional connectomics from resting-state fMRI. Trends Cogn Sci. (2013) 17:666–82. 10.1016/j.tics.2013.09.016 24238796 PMC4004765

[34] Fornito A Zalesky A Bullmore E. Fundamentals of Brain Network Analysis. Cambridge, MA: Academic Press (2016).

[35] Su H Zhang L Qiao L Liu M. Estimating high-order brain functional networks by correlation-preserving embedding. Med Biol Eng Comput. (2022) 60:2813–23. 10.1007/s11517-022-02628-7 35869385

[36] Meinshausen N Bühlmann P. High-dimensional graphs and variable selection with the lasso. Ann Stat. (2006) 34:1436–62. 10.1214/009053606000000281

[37] Friedman J Hastie T Tibshirani R. Sparse inverse covariance estimation with the graphical lasso. Biostatistics. (2008) 9:432–41. 10.1093/biostatistics/kxm045 18079126 PMC3019769

[38] Blei DM Kucukelbir A McAuliffe JD. Variational inference: a review for statisticians. J Am Stat Assoc. (2017) 112:859–77. 10.1080/01621459.2017.1285773

[39] Wee CY Yap PT Zhang D Wang L Shen D. Group-constrained sparse fMRI connectivity modeling for mild cognitive impairment identification. Brain Struct Funct. (2014) 219:641–56. 10.1007/s00429-013-0524-8 23468090 PMC3710527

[40] Chang CC Lin CJ. LIBSVM: a library for support vector machines. ACM Trans Intell Syst Technol. (2011) 2:1–27. 10.1145/1961189.1961199

[41] Jiang X Zhang L Qiao L Shen D. Estimating functional connectivity networks via low-rank tensor approximation with applications to MCI identification. IEEE Trans Biomed Eng. (2019) 67:1912–20. 10.1109/TBME.2019.2950712 31675312

[42] Sun Y Yao X March ME Meng X Li J Wei Z. Target genes of autism risk loci in brain frontal cortex. Front Genet. (2019) 10:707. 10.3389/fgene.2019.00707 31447881 PMC6696877

[43] Mouga S Duarte IC Café C Sousa D Duque F Oliveira G. Parahippocampal deactivation and hyperactivation of central executive, saliency and social cognition networks in autism spectrum disorder. J Neurodev Disord. (2022) 14:1–12. 10.1186/s11689-022-09417-1 35078414 PMC8903486

[44] Turner AH Greenspan KS van Erp TG. Pallidum and lateral ventricle volume enlargement in autism spectrum disorder. Psychiatry Res Neuroimaging. (2016) 252:40–5. 10.1016/j.pscychresns.2016.04.003 27179315 PMC5920514

[45] Monk CS Peltier SJ Wiggins JL Weng SJ Carrasco M Risi S. Abnormalities of intrinsic functional connectivity in autism spectrum disorders. Neuroimage. (2009) 47:764–72. 10.1016/j.neuroimage.2009.04.069 19409498 PMC2731579

[46] Cheng Y Chou KH Chen IY Fan YT Decety J Lin CP. Atypical development of white matter microstructure in adolescents with autism spectrum disorders. Neuroimage. (2010) 50:873–82. 10.1016/j.neuroimage.2010.01.011 20074650

[47] Van Rooij D Anagnostou E Arango C Auzias G Behrmann M Busatto GF. Cortical and subcortical brain morphometry differences between patients with autism spectrum disorder and healthy individuals across the lifespan: results from the ENIGMA ASD Working Group. Am J Psychiatry. (2018) 175:359–69. 10.1176/appi.ajp.2017.17010100 29145754 PMC6546164

